# Function-based personalization of dietary fiber selection enhances gut microbial fermentation: a proof-of-concept crossover trial

**DOI:** 10.3389/fnut.2026.1907988

**Published:** 2026-09-03

**Authors:** Ro Osawa, Jun Inoue, Itsuko Fukuda, Yasuhito Shirai, Yuzo Kodama

**Affiliations:** 1 Division of Gastroenterology, Department of Internal Medicine, Graduate School of Medicine, Kobe University, Kobe, Japan; 2 Research Center for Food Safety and Security, Graduate School of Agricultural Science, Kobe University, Kobe, Japan

**Keywords:** dietary fiber, *ex vivo* fermentation, gut microbiome, personalized nutrition, prebiotics, precision nutrition, short-chain fatty acids

## Abstract

Responses to dietary fiber supplementation vary markedly among individuals, yet no validated platform exists to prospectively identify optimal fiber types based on individual gut microbiome function. Here we present My Dietary Fiber Finder (MyDFF), an *ex vivo* functional screening platform that predicts optimal dietary fibers for individual gut microbiomes through direct fermentation profiling. For each participant, the dietary fiber with the highest saccharification index (SI) was designated M-PDF, and that with the lowest SI was designated L-PDF. In a crossover trial involving eight healthy Japanese adults (72 longitudinal observations, each participant serving as their own control), the pre-specified per-participant analysis (Dunnett’s test) showed that M-PDF significantly increased fecal volatile fatty acid (VFA; acetate + propionate + butyrate) concentrations over baseline in two of eight participants and total short-chain fatty acid (SCFA) concentrations in one, whereas the non-personalized fiber (L-PDF) did so in none. *Post hoc* group-level analysis confirmed a significant increase over baseline for M-PDF (total VFA and total SCFA, both *p* < 0.01) but not for L-PDF, and in a within-participant comparison M-PDF exceeded L-PDF in seven of eight participants (mean difference = 32.4 μmol/g; 95% CI: 8.7–56.1; *p* = 0.014; Cohen’s d = 1.14). However, the magnitude of the pre-intervention saccharification-index differential between the two fibers did not significantly correlate with the magnitude of the *in vivo* VFA response differential across participants (Pearson *r* = 0.082, *p* = 0.85), indicating that the assay did not quantitatively predict response magnitude. Exploratory 16S rRNA gene sequencing analysis showed no significant cohort-level shift in overall gut microbial community composition during either intervention phase. In four separate validation donors, individual fermentation profiles remained relatively stable over the observed 3–9-month follow-up. These proof-of-concept findings support the potential of function-based framework for personalized prebiotic design and motivate larger validation studies.

**Clinical trial registration:**

https://jrct.niph.go.jp, identifier jRCTs051220163.

## 1 Introduction

The human gut microbiome ferments dietary fiber into short-chain fatty acids (SCFAs)—predominantly acetate, propionate, and butyrate. Because these metabolites are volatile under standard analytical conditions, they are often measured and reported as volatile fatty acids (VFAs); throughout this paper, we use VFA when referring to our fecal measurements and SCFA in the broader physiological context. These metabolites function as essential signaling molecules regulating epithelial barrier integrity, immune homeostasis, and systemic metabolism (1, 2). SCFA production has been linked to protection against metabolic syndrome, inflammatory diseases, and colorectal cancer, establishing gut microbial fermentation as a critical node in diet–health interactions (3–6).

Contemporary dietary patterns in developed nations are characterized by chronically insufficient fiber intake, stimulating considerable interest in prebiotic supplementation strategies (7–9). However, clinical outcomes of prebiotic interventions exhibit marked inter-individual variability, with responses ranging from substantial metabolic improvements to minimal effect (10–12). This heterogeneity reflects complex interactions among host genetics, microbial community composition, and environmental factors (13–18). Critically, baseline microbiome composition can help explain and, in some settings, predict why individuals differ in their responses to a given dietary fiber (14, 17, 19, 20). However, the relative fermentation responses to different fibers can vary substantially within the same individual (12, 15–17), and current taxonomic approaches have not been shown to reliably determine in advance which of several candidate fibers will be optimal for that individual. Accordingly, no validated platform has yet been established to prospectively compare multiple candidate fibers and identify the optimal fiber for an individual microbiome before intervention (19, 20).

The predominant reliance on uniform fiber recommendations inadequately addresses individual microbiome functional capacity, potentially explaining the inconsistent clinical outcomes observed across prebiotic trials. Approaches based on taxonomic profiling alone have proven insufficient predictors of functional metabolic responses (21, 22).

Prior personalized prebiotic studies have demonstrated retrospectively that individual identity is a stronger determinant of SCFA response than fiber type (15, 23), and that taxonomic profiles can predict responses to specific fibers. However, these approaches identify responders after, rather than prospectively guiding fiber selection before, intervention. Moreover, they rely predominantly on taxonomic composition to stratify individuals, despite growing evidence that functional capacity does not map directly onto compositional profiles (12). MyDFF addresses both limitations by measuring each individual’s gut microbiota fermentation capacity directly and prospectively, enabling function-based fiber matching prior to intervention.

Here, we present MyDFF, a function-based screening platform that prospectively identifies optimal dietary fibers for individual gut microbiomes using a modified version of our previously established human intestinal model (24–27). In a proof-of-concept crossover trial with sequential (enrollment-order) allocation, we tested whether this approach enhances *in vivo* fecal VFA production, hypothesizing that the MyDFF-selected fiber would elicit greater fermentation than a non-personalized fiber within the same individual.

## 2 Materials and methods

### 2.1 MyDFF dietary fiber panel and *ex vivo* fermentation assay

The dietary fiber panel comprised 17 commercially available supplements representing six categories: resistant starch (*n* = 4), resistant dextrin (*n* = 3), *α*-cyclodextrin (*n* = 3), isomaltodextrin (*n* = 2), partially hydrolyzed guar gum (*n* = 2), and inulin (*n* = 3); full product details are provided in Supplementary Table S1. Individual fibers were added at 1% (w/v) final concentration to 1 mL of modified Gifu Anaerobic Medium (28) in 96-well format. Each well was inoculated with 50 μL of fecal suspension (~10^8^ cells/mL) and incubated anaerobically (AnaeroPack system; Mitsubishi Gas Chemical Company, Tokyo, Japan) at 37 °C for 24 h. Post-incubation pH was measured using a microelectrode (ToupH 9,669-10D; HORIBA Advanced Techno, Kyoto, Japan). All fermentations were performed in triplicate. The saccharification index (SI) was calculated as the mean ΔpH between fiber-supplemented and control wells. In this study, the “optimal” fiber for each participant was operationally defined as the fiber eliciting the greatest saccharolytic response—that is, the largest SI—in that individual’s *ex vivo* fecal microbiota assay. SI was used to rank candidate fibers within an individual; it correlates with total SCFA output at the assay level (Supplementary Figure S1) but is not intended as a quantitative predictor of the magnitude of an individual’s *in vivo* response, nor does it imply optimization of a specific SCFA fraction or clinical outcome.

The saccharification index (SI) was used as the primary fermentation readout for the following reasons. Biochemically, the primary metabolic end-products of anaerobic fermentation of dietary fiber by gut microbiota are VFAs (acetate, propionate, butyrate) and minor organic acids; these are the dominant proton donors responsible for culture medium acidification, so the magnitude of pH decrease directly tracks fermentative activity. The pH-based approach was selected over direct HPLC quantification in the screening phase to enable high-throughput, parallel assessment of up to 17 fiber substrates per individual (up to 51 wells per person) at clinical scale, which would be cost-prohibitive with HPLC analysis. This approach follows established precedent in intestinal fermentation model systems (24–27). To confirm empirically that SI reliably reflects SCFA production under our assay conditions, spent fermentation media from 30 randomly selected wells (fecal *ex vivo* fermentation cultures from six healthy adults) spanning the full range of observed pH changes were analyzed by HPLC. SI values showed a strong positive correlation with total SCFA concentrations (*r* = 0.89, r^2^ = 0.79; Supplementary Figure S1), with the 95% confidence interval of the regression demonstrating consistent predictive accuracy across the full range of fermentation intensities. The fiber eliciting the highest mean SI across three replicate assays was designated M-PDF; the fiber with the lowest mean SI was designated L-PDF. When two fibers produced identical mean SI values, the fiber with the smaller standard deviation was preferred.

### 2.2 Validation study: sample collection and participant ethics

This cross-sectional and longitudinal analysis was approved by the Institutional Ethics Review Board of Kobe University (Approval No. 1902) and conducted in accordance with the Declaration of Helsinki. Written informed consent was obtained from all participants. Fresh fecal samples were collected from 10 healthy adult donors (aged 20–70 years; designated A–J) to evaluate interindividual differences in responses to each dietary fiber. A subset of four donors (B, C, I, J) provided follow-up samples at one month and either three or 9 months post-baseline to assess longitudinal profile stability.

### 2.3 Intervention study design and participant ethics

This exploratory proof-of-concept dietary intervention study was prospectively registered in the Japan Registry of Clinical Trials (jRCTs051220163) and approved by the Institutional Ethics Review Board of Kobe University (Approval No. C220007; approved 12 December 2022). All participants provided written informed consent. Eight healthy adults (aged 34–62 years) were enrolled; exclusion criteria included diabetes mellitus, liver, kidney, cardiac, or pulmonary disease; any current medical condition requiring treatment; known food or drug allergies.

**Figure 1:**
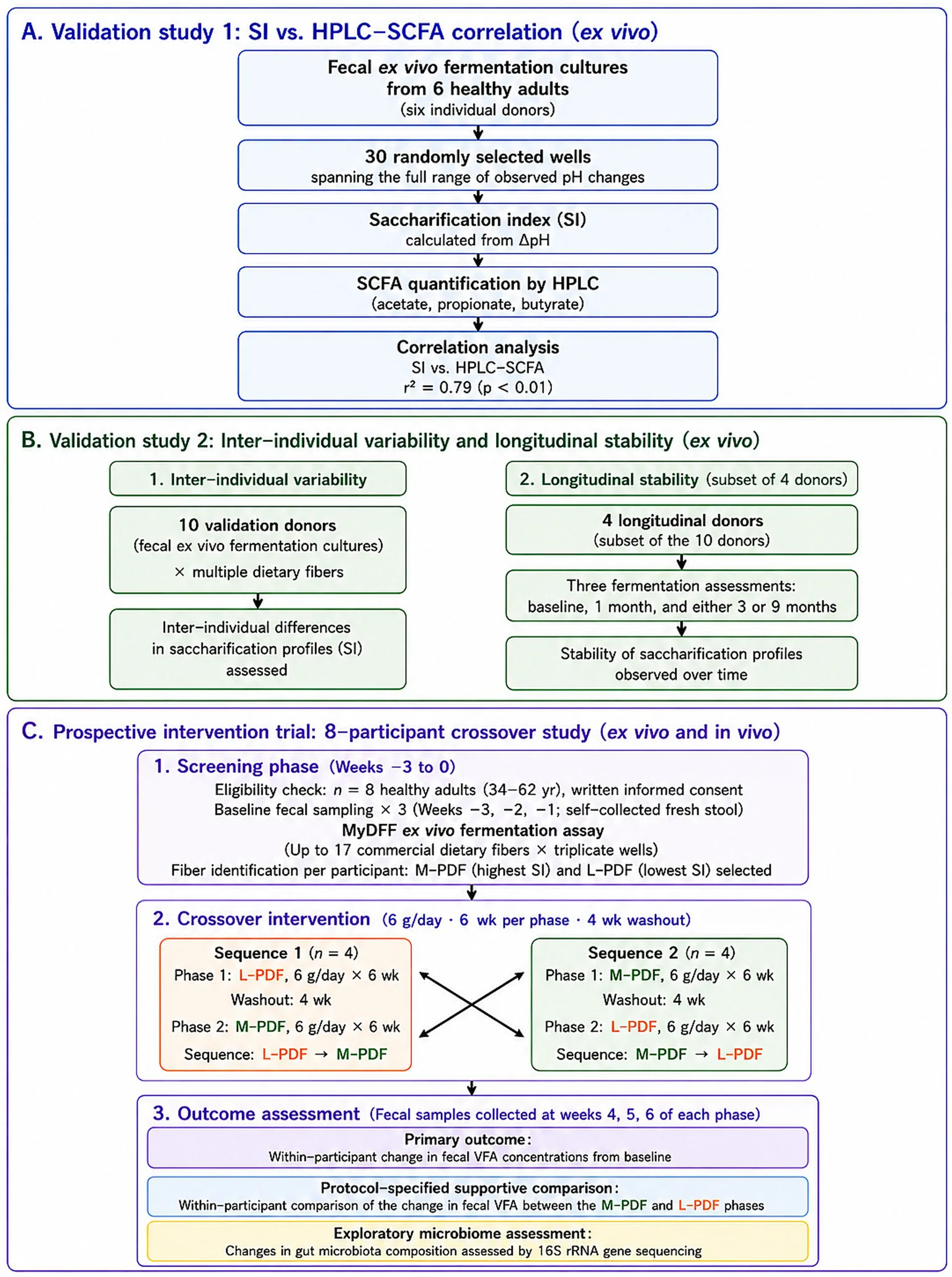
Overview of the validation studies and prospective intervention trial. **(A)** Validation study 1: Validation of the saccharification index against HPLC-quantified SCFAs. To empirically confirm that the saccharification index (SI) reflected SCFA production under the MyDFF assay conditions, spent fermentation media from 30 randomly selected wells derived from fecal *ex vivo* fermentation cultures of six healthy adults, spanning the full range of observed pH changes, were analyzed by HPLC. SI was correlated with HPLC-quantified SCFA concentrations (*r*^2^ = 0.79, *p* < 0.01). **(B)** Validation study 2: Inter-individual variability and longitudinal stability. Saccharification profiles were evaluated in 10 validation donors to assess inter-individual differences. Four of these donors underwent three fermentation assessments—at baseline, 1 month, and either 3 or 9 months—to evaluate the longitudinal stability of individual saccharification profiles. **(C)** Prospective intervention trial. Eight healthy adults (34–62 years) provided written informed consent and underwent baseline fecal sampling at weeks −3, −2, and −1. MyDFF *ex vivo* fermentation assays were performed using up to 17 commercial dietary fibers in triplicate wells, and the fibers yielding the highest and lowest SI for each participant were designated M-PDF and L-PDF, respectively. Participants underwent a two-period crossover intervention (6 g/day for 6 weeks per phase, with a 4-week washout). Sequence allocation was based on enrollment order rather than randomization: Participants Nos. 1, 3, 5, and 7 received L-PDF followed by M-PDF, whereas Nos. 2, 4, 6, and 8 received M-PDF followed by L-PDF, resulting in a balanced 4:4 allocation. Fecal samples were collected at weeks 4, 5, and 6 of each intervention phase. The primary outcome was the within-participant change in fecal VFA concentrations from baseline. A protocol-specified supportive comparison evaluated the within-participant difference in VFA response between the M-PDF and L-PDF phases. Exploratory microbiome assessment evaluated changes in gut microbiota composition by 16S rRNA gene sequencing.

We employed a single-blind crossover design with sequential allocation by enrollment order (rather than formal randomization), comprising a 3-week baseline period, two 6-week intervention periods separated by a 4-week washout (Figure 1). Participants were allocated by enrollment order: odd-numbered participants (*n* = 4) received L-PDF followed by M-PDF; even-numbered participants (*n* = 4) received the reverse. Participants consumed 6 g of assigned fiber daily (3 g twice daily with meals) in identical opaque containers with neutral labeling.

### 2.4 Monitoring of dietary intake, medication, and potential confounders

To capture host and environmental factors known to influence fecal VFA production, participants were monitored throughout the study using periodic physician-administered interviews (Figure 1). The attending physician conducted structured interviews at multiple timepoints in each period, in conjunction with a symptom questionnaire to assess intercurrent illness, medication, and health-food/supplement use, and adherence to the assigned study dietary fiber. Concomitant medication and illness were monitored across the entire study period, including baseline and washout. Because the crossover design has each participant serve as their own control, stable inter-individual differences in baseline microbiome composition and habitual lifestyle are inherently accounted for, and the 4-week washout further minimized carryover. We did not systematically quantify total habitual dietary intake or physical activity; monitoring was designed to capture deviations relevant to gut fermentation (fiber and probiotic supplements, antibiotics, and gastrointestinal medications) rather than full dietary assessment.

### 2.5 Sample size and power

The sample size was pre-specified in the registered protocol (jRCTs051220163). Unpublished preliminary measurements indicated that the fecal short-chain fatty acid concentration in repeated samples from the same individual was approximately 100 ± 10 μmol/g (mean ± SD). The baseline concentration was therefore assumed to be 100 ± 10 μmol/g, and supplementation with the fiber producing the greatest pH decrease in the MyDFF assay was assumed to raise it to 135 ± 10 μmol/g. Under these assumptions, a two-sided paired *t*-test at a significance level (*α*) of 0.05 and a type II error rate (*β*) of 0.20 (80% power) indicated that eight participants would be sufficient. The crossover design, in which each participant serves as their own control, reduces the influence of stable between-participant variability on the primary comparison (29) and is well suited to pilot studies with intensive longitudinal sampling. As a prospectively designed exploratory proof-of-concept study with an intensive protocol (19 weeks of participation with multiple stool collections per participant), we set this as the minimum sample size that we judged sufficient, on scientific and feasibility grounds, to generate preliminary evidence while remaining feasible to complete.

### 2.6 SCFA/VFA quantification by HPLC

Thawed fecal samples were processed and SCFAs/VFAs were quantified by HPLC using an Aminex HPX-87H ion-exclusion column with refractive index detection (RID-10A; Shimadzu Corporation, Kyoto, Japan). Full details of sample preparation and chromatographic conditions are provided in Supplementary methods.

### 2.7 16S rRNA gene sequencing

Bacterial genomic DNA was extracted from thawed fecal samples using the QIAamp PowerFecal Pro DNA Kit (Qiagen), which incorporates a bead-beating step, as described previously (24). The V3–V4 hypervariable regions were amplified using universal primers S-D-Bact-0341-b-S-17 and S-D-Bact-0785-a-A-21. Paired-end sequencing (2 × 300 bp) was performed on an Illumina MiSeq platform using MiSeq Reagent Kit v3 with PhiX Control v3 as an internal sequencing control. Demultiplexed paired-end reads were imported into QIIME 2 (v2025.7) and processed using DADA2.

Among the 72 samples included in the microbiome analyses, the number of input read pairs ranged from 81,386 to 1,401,550 per sample (mean, 151,684; median, 133,526). Denoising parameters were as follows: trim-left 0/0; trunc-len 285/230; trunc-q 2; maxEE 2.0/3.0; n-reads-learn 10,000. Following quality filtering, denoising, paired-end merging, and consensus chimera removal, 5,800–75,361 non-chimeric sequences per sample remained (mean, 12,692; median, 11,989). The final feature table contained 6,419 DADA2-generated amplicon sequence variants (ASVs). No samples were excluded because of sequencing failure, DADA2 quality-control failure, or insufficient sequencing depth. For phylogenetic analyses, representative ASVs were aligned with MAFFT, hypervariable positions were masked, and a midpoint-rooted FastTree was constructed within QIIME 2. Taxonomy was assigned using q2-feature-classifier with a pre-trained naïve Bayes classifier trained on the SILVA version 138.1 SSURef NR99 reference dataset (confidence threshold 0.7); no fixed sequence-similarity threshold was applied, consistent with the exact-sequence resolution of DADA2-generated ASVs. The rarefaction depth of 5,000 sequences per sample was selected because the minimum post-DADA2 sequence count across all 72 samples was 5,800 (allowing all samples to be retained), and because alpha-rarefaction curves showed diminishing gains in the principal alpha-diversity metrics at approximately this depth.

### 2.8 Statistical analyses

The primary endpoint was the per-participant change in fecal VFA concentrations between baseline and each intervention phase, as specified in the statistical analysis plan. For each participant, the three fecal samples collected per phase were compared across phases (baseline, L-PDF, M-PDF) by one-way ANOVA with Dunnett’s multiple-comparison test (L-PDF vs. baseline; M-PDF vs. baseline) in Statcel2; this was performed for both total VFA and total SCFA. The within-participant contrast between the two intervention phases (M-PDF vs. L-PDF ΔVFA), which was specified in the study protocol, was assessed by paired *t*-test with the Wilcoxon signed-rank test as a robustness check. As a *post hoc* supportive analysis, baseline-referenced contrasts (L-PDF vs. baseline; M-PDF vs. baseline) for total VFA were evaluated with a linear mixed-effects model fitted to all 72 measurements, with study phase as a fixed effect and participant as a random intercept (restricted maximum likelihood; Kenward–Roger degrees of freedom; emmeans), with the two baseline-referenced contrasts adjusted by a Dunnett-type procedure with multivariate-t adjustment. The probability of observing seven of eight participants responding in the same direction by chance was assessed in a *post hoc* exploratory analysis using a two-sided binomial test. Group-level beta-diversity differences were evaluated by PERMANOVA. Hierarchical cluster analysis of prebiotic response spectra used the UPGMA algorithm with the Horn similarity index (30, 31) implemented in R (v4.3.0). All tests were two-sided; *p* < 0.05 was considered statistically significant. Effect sizes are reported as Cohen’s d.

Multiple comparison considerations: (1) The primary per-participant baseline-referenced comparisons (L-PDF vs. BL; M-PDF vs. BL) used Dunnett’s test (Statcel2), which controls the family-wise error rate across the two simultaneous comparisons against the common baseline within each participant. (2) The protocol-specified within-participant comparison of M-PDF versus L-PDF ΔVFA constitutes a single comparison and therefore requires no further multiplicity adjustment. (3) The per-participant alpha-diversity comparisons (Chao1, Shannon, observed ASVs) are exploratory: because they comprise a large number of individual-level tests in a small cohort, we report nominal (uncorrected) *p*-values and interpret them descriptively rather than applying a formal family-wise correction. The cohort-level assessment of whether community structure shifted was based on the group-level PERMANOVA of weighted UniFrac distances.

## 3 Results

### 3.1 Validation of the MyDFF platform

SI values showed strong positive correlation with total SCFA concentrations measured by HPLC across 30 randomly selected fermentation wells (fecal *ex vivo* fermentation cultures from six healthy adults: *r* = 0.89, r^2^ = 0.79, *p* < 0.01; Supplementary Figure S1), confirming that pH-based measurements reliably reflect microbial fermentation capacity. Analysis of baseline fecal samples from ten validation donors (A–J) revealed substantial inter-individual variation in prebiotic response profiles (Supplementary Figure S2).

**Figure 2:**
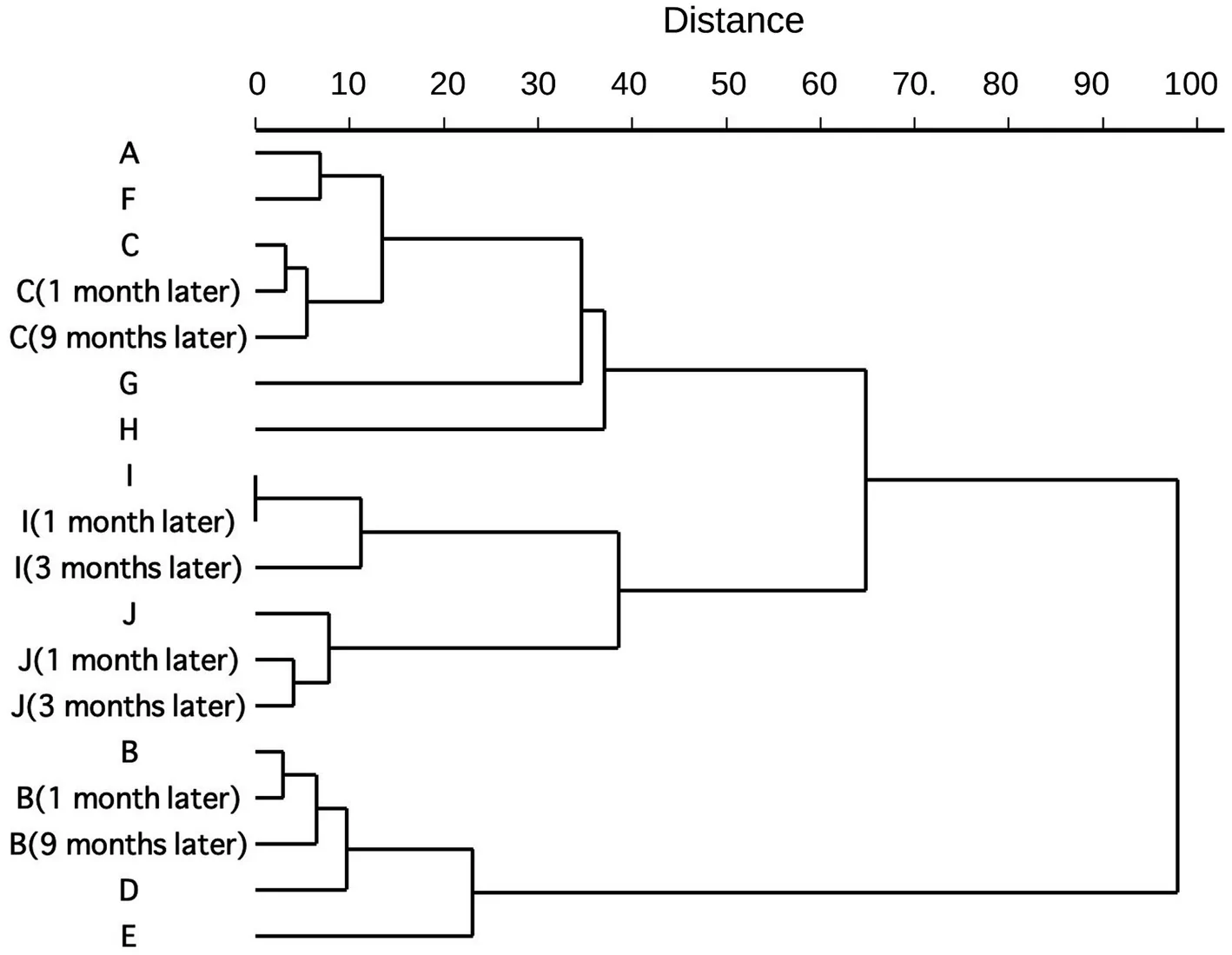
Temporal stability of individual prebiotic response profiles. UPGMA dendrogram of prebiotic response spectra from ten validation donors (A–J). Longitudinal samples from donors B, C, I, and J were collected at baseline and follow-up timepoints (1 month for all four; 3 months for I and J; 9 months for B and C). Distance values represent dissimilarity calculated using the Horn similarity index. Repeated samples from the same individual cluster tightly (mean within-individual distance = 4.2 ± 1.8), clearly separated from other individuals (mean between-individual distance = 38.5 ± 15.3), demonstrating temporal stability of functional fermentation profiles over 3–9 months.

Hierarchical cluster analysis of longitudinal samples from four donors (B, C, I, J) demonstrated that repeated samples from the same individual consistently clustered together (mean within-individual dissimilarity distance = 4.2 ± 1.8), clearly separated from other individuals (mean between-individual distance = 38.5 ± 15.3; Figure 2). In the four longitudinally followed validation donors, individual prebiotic response profiles remained relatively similar at the observed follow-up timepoints (1 month and either 3 or 9 months; Supplementary Figure S3), suggesting temporal stability over the observed interval in this small validation subset.

### 3.2 Intervention study: participant characteristics and completion

The study was conducted between August 2023 and April 2025. The median participant age was 42.5 years (range: 34–62 years), with a male-to-female ratio of 6:2. All eight enrolled participants completed the full protocol with 100% retention. MyDFF analysis of baseline fecal samples identified personalized M-PDF and L-PDF pairs for each participant. The M-PDF was inulin for six participants (Nos. 1, 2, 4, 5, 6, and 7), *α*-cyclodextrin for one (No. 3), and isomaltodextrin for one (No. 8).

### 3.3 Intercurrent exposures during the intervention

Records from nine physician interviews per participant were reviewed for all eight participants (Supplementary Table S4). Reported exposures were infrequent and mostly comprised occasional over-the-counter analgesics or vitamin supplements with no established effect on colonic fermentation. Exposures of potential relevance to microbial fermentation were identified in two participants. Both received a 1-week course of the same oral cephalosporin antibiotic (cefcapene pivoxil) for unrelated reasons, in different study periods: participant 7 during the washout, and participant 8 during the pre-observation period (with the baseline MyDFF screening performed after the course had ended). Neither course overlapped the fecal-sampling windows used for VFA quantification. Participant 7 was among the seven responders, whereas participant 8 was the sole non-responder; because the two antibiotic-exposed participants differed in outcome and both exposures lay outside the sampling windows, the available observations do not suggest a uniform directional association between antibiotic exposure and response; however, residual confounding cannot be excluded. Participant 8 additionally consumed probiotic fermented-dairy products intermittently; with a single non-responder, we cannot determine whether the pre-observation antibiotic (which could have influenced the baseline screening), the probiotic intake, or genuine biological non-response accounts for the absence of an M-PDF advantage. No participant reported use of dietary-fiber supplements other than the assigned product during the intervention phases.

### 3.4 MyDFF-guided supplementation enhances VFA production

We next examined whether MyDFF-guided fiber selection increases fecal VFA concentrations *in vivo*. In the pre-specified primary analysis, VFA and SCFA concentrations were compared against baseline within each participant by Dunnett’s test (Statcel2). At this individual level, the M-PDF phase significantly exceeded baseline for total VFA in two of the eight participants (participant 3, *p* < 0.01; participant 5, *p* < 0.05) and for total SCFA in one participant (participant 3, *p* < 0.01), whereas the L-PDF phase did not reach significance in any participant for either measure. Individual-level significance was thus confined to the MyDFF-selected fiber, although the three samples available per phase limit the power of this within-participant test.

**Figure 3:**
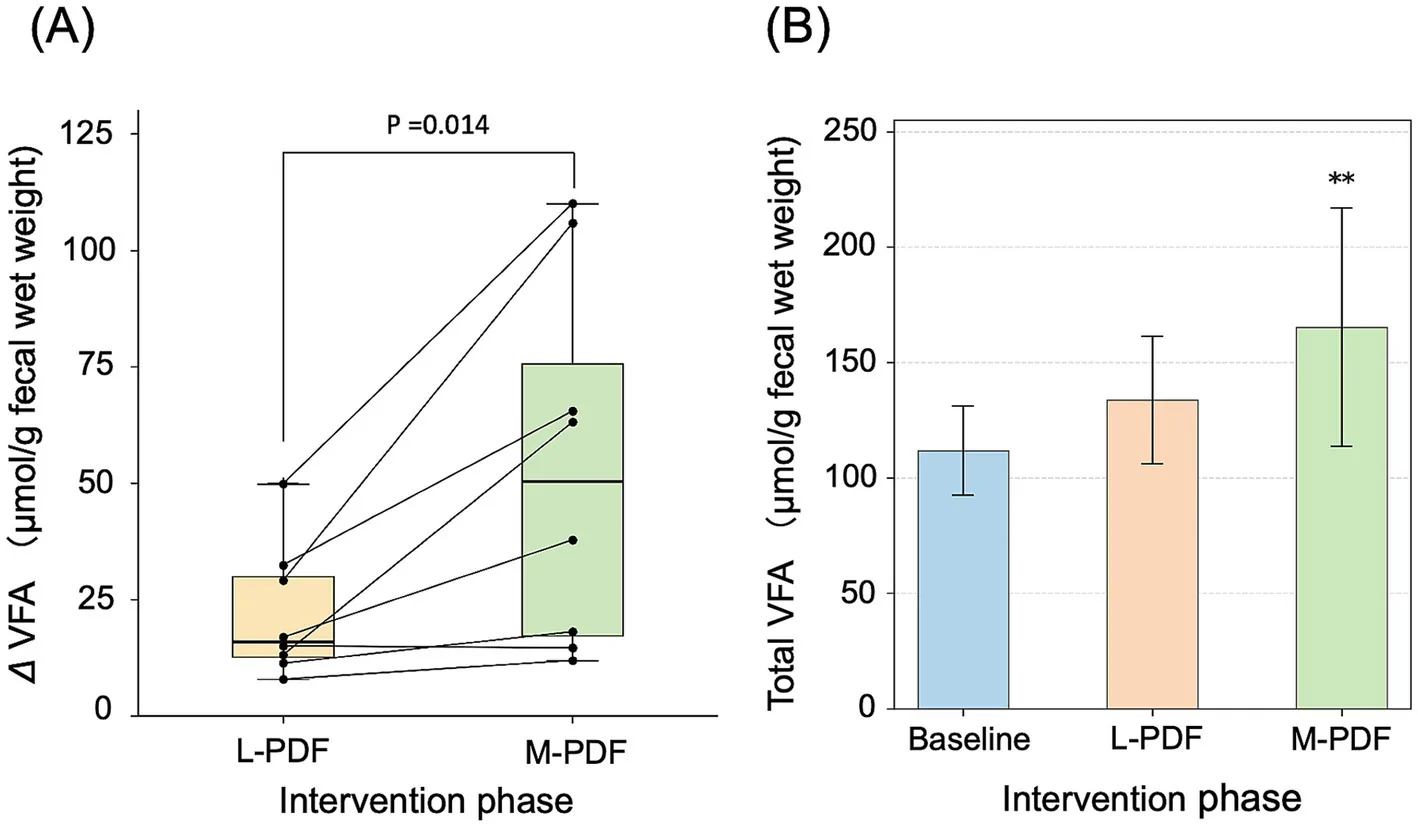
MyDFF-guided fiber supplementation enhances fecal VFA production. **(A)** Within-participant changes in VFA from baseline (ΔVFA, μmol/g fecal wet weight) during the L-PDF and M-PDF phases (*n* = 8). Boxes show the median (centre line) and interquartile range; whiskers span the full range. Lines connect paired observations from the same participant. Seven of the eight participants showed a larger increase during M-PDF; in the remaining participant the two phases were essentially equivalent. Protocol-specified within-participant comparison by paired *t*-test (mean difference = 32.4 μmol/g; 95% CI: 8.7–56.1; *p* = 0.014; Wilcoxon *p* = 0.016; Cohen’s *d*_z = 1.14). **(B)** Total fecal VFA concentrations during the baseline (BL), L-PDF, and M-PDF phases. Bars show the mean ± SD across *n* = 8 participants; each participant value is the mean of three measurements within that phase. Asterisks (**) denote a significant increase over baseline in a *post hoc* baseline-referenced linear mixed-effects model with Dunnett-type adjustment: M-PDF vs. baseline, *p* < 0.0001; L-PDF vs. baseline was not significant (*p* = 0.104). Total SCFA showed the same pattern.

In the protocol-specified within-participant comparison of the two intervention phases, ΔVFA during the M-PDF phase exceeded that during the L-PDF phase by 32.4 μmol/g on average (95% CI: 8.7–56.1; paired-*t p* = 0.014; Wilcoxon *p* = 0.016; Cohen’s d = 1.14; Figure 3A). Seven of the eight participants showed a greater ΔVFA during the M-PDF phase, whereas the remaining participant (No. 8) showed nearly identical increases during the two phases (ΔVFA = +14.5 vs. + 15.0 μmol/g). Individual ΔVFA during the M-PDF phase ranged from 11.9 to 110.1 μmol/g, indicating substantial inter-individual variation in the magnitude of response.

As a *post hoc* supportive analysis, baseline-referenced contrasts were evaluated with a linear mixed-effects model fitted to all 72 fecal VFA measurements (study phase as a fixed effect, participant as a random intercept) with Dunnett-type adjustment. Total fecal VFA was significantly higher during the M-PDF phase than at baseline (estimated mean difference, 54.4 μmol/g; simultaneous 95% CI, 28.7 to 80.1; Dunnett-adjusted *p* < 0.0001), whereas the L-PDF phase did not differ significantly from baseline (estimated mean difference, 21.9 μmol/g; simultaneous 95% CI, −3.8 to 47.6; Dunnett-adjusted *p* = 0.104) (Figure 3B). Thus, at both the individual and cohort levels, a significant increase over baseline was confined to the MyDFF-selected fiber. In a *post hoc* exploratory analysis, the directional concordance observed in seven of eight participants did not reach statistical significance in a two-sided exact binomial test (*p* = 0.070).

Individual SCFA and VFA concentrations across all phases are summarized in Supplementary Table S2.

### 3.5 Association between *ex vivo* and *in vivo* responses

The SI differential (ΔSI = SI_M-PDF − SI_L-PDF) calculated from baseline measurements was not significantly associated with the magnitude of the VFA response differential (ΔVFA = VFA_M-PDF − VFA_L-PDF) measured during intervention phases (Pearson *r* = 0.082, *p* = 0.85; Table 1). This near-zero correlation was strongly influenced by participant 8, who had the largest predicted differential (ΔSI = 1.00) yet showed no *in vivo* response and was the sole non-responder. Thus, although seven of eight participants showed a greater VFA increase during the M-PDF phase than during the L-PDF phase, the magnitude of the *ex vivo* SI differential did not quantitatively predict the magnitude of the *in vivo* VFA response differential in this small cohort. Host factors known to influence fecal VFA—including medication use and intake of other fiber or probiotic products—were prospectively monitored, and baseline microbiome and lifestyle differences are controlled by the within-participant crossover design (see Materials and Methods, “Monitoring of dietary intake, medication, and potential confounders”; Supplementary Table S4).

**Table 1:** Individual saccharification indices and VFA production across intervention phases.

Participant No.	Intervention phase	DF supplemented	Mean SI (L-PDF fiber)	Mean SI (M-PDF fiber)	Total VFA (μmol/g)	Total SCFA (μmol/g)
1	BL	None	0.41	1.01	132.9	135.1
	L-PDF	Partially hydrolyzed guar gum	0.59	0.88	146.0	153.1
	M-PDF	Inulin	0.66	1.01	196.1	200.6
2	BL	None	0.07	1.01	111.1	111.1
	L-PDF	Partially hydrolyzed guar gum	0.31	0.88	140.3	147.4
	M-PDF	Inulin	0.11	1.01	217.1	229.6
3	BL	None	0.00	0.70	116.5	118.5
	L-PDF	Partially hydrolyzed guar gum	0.27	0.80	166.4	166.4
	M-PDF	α-cyclodextrin	0.17	0.70	226.6	226.6
4	BL	None	0.33	1.01	108.8	108.8
	L-PDF	Resistant starch	0.41	0.95	125.7	125.7
	M-PDF	Inulin	0.41	0.92	154.3	154.3
5	BL	None	0.23	0.98	138.2	153.1
	L-PDF	Partially hydrolyzed guar gum	0.26	0.88	170.6	170.6
	M-PDF	Inulin	0.18	0.93	203.7	209.2
6	BL	None	0.13	0.76	115.7	115.7
	L-PDF	Resistant starch	0.39	0.86	123.6	129.1
	M-PDF	Inulin	0.16	1.12	127.6	133.1
7	BL	None	0.35	1.00	80.7	80.7
	L-PDF	Partially hydrolyzed guar gum	0.47	0.49	92.0	92.0
	M-PDF	Inulin	0.48	0.81	98.8	98.8
8	BL	None	0.04	1.04	90.6	90.6
	L-PDF	Resistant starch	0.02	0.87	105.6	105.6
	M-PDF	Isomaltodextrin	0.07	0.67	105.1	105.1

Mean SI values for the L-PDF and M-PDF fibers identified for each participant by MyDFF screening, together with fecal VFA and SCFA concentrations during baseline (BL), L-PDF, and M-PDF intervention phases. The SI differential (ΔSI = SI_M-PDF–SI_L-PDF) did not significantly predict the VFA response differential (ΔVFA = VFA_M-PDF–VFA_L-PDF) across participants (Pearson *r* = 0.082, *p* = 0.85; *r* = 0.65, *p* = 0.11 excluding the sole non-responder, participant 8). SI, saccharification index (mean ΔpH from triplicate *ex vivo* MyDFF assays); Higher values indicate greater fermentation capacity. Total VFA, sum of acetate, propionate, and butyrate; Total SCFA, total VFA plus lactate and succinate, quantified by HPLC (mean of triplicate fecal samples, weeks 4–6 of each phase). DF, dietary fiber; BL, baseline; L-PDF/M-PDF, least/most prebiotic dietary fiber.

### 3.6 Gut community composition is largely stable during intervention

**Figure 4:**
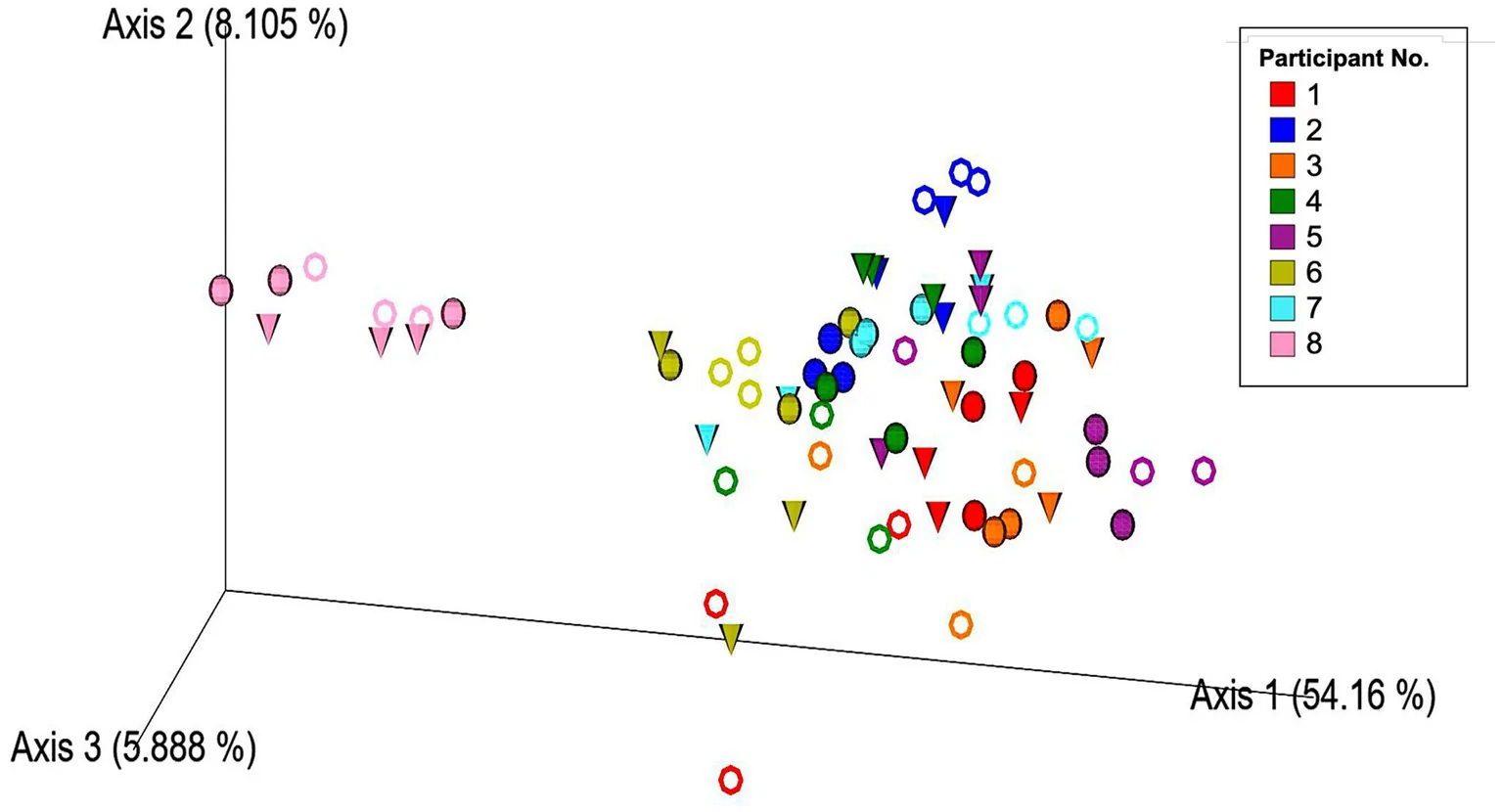
Gut microbial community structure across dietary intervention phases. Three-dimensional PCoA plot of gut microbial *β*-diversity based on weighted UniFrac distances for eight participants (color-coded by participant number) during baseline (open circles), M-PDF (filled circles), and L-PDF (inverted triangles) phases. Samples cluster by participant identity rather than intervention phase. PERMANOVA: baseline vs. L-PDF, *p* = 0.615; baseline vs. M-PDF, *p* = 0.753. Axes represent the percentage of variance explained.

PERMANOVA analysis revealed no significant group-level community structure shifts following either L-PDF (*p* = 0.615) or M-PDF (*p* = 0.753) supplementation. PCoA of weighted UniFrac distances demonstrated that inter-individual variation substantially exceeded intervention-induced changes, with samples clustering primarily by participant identity rather than intervention phase (Figure 4). Pairwise PERMANOVA comparisons among all participant pairs confirmed that participant identity was the dominant source of compositional variation, with all 28 comparisons remaining significant after false-discovery-rate correction (q < 0.05; Supplementary Table S3).

It should be noted that 16S rRNA sequencing characterizes taxonomic community composition (who is present) rather than metabolic function. In this study, functional performance (VFA/SCFA production) was measured directly and independently by HPLC, which provided the functional measurement for the primary endpoint. The role of 16S sequencing was strictly to determine whether major restructuring of community composition accompanied the functional changes. The conclusion that functional enhancement occurred without major compositional restructuring therefore rests on two independent measurements: HPLC-measured VFA production (function) and PERMANOVA on 16S data (composition). Deeper mechanistic resolution—identifying specific taxa, strains, or enzymatic pathways responsible for fiber-specific fermentation responses—would require metatranscriptomic or metagenomic approaches, identified here as a priority for future work.

**Figure 5:**
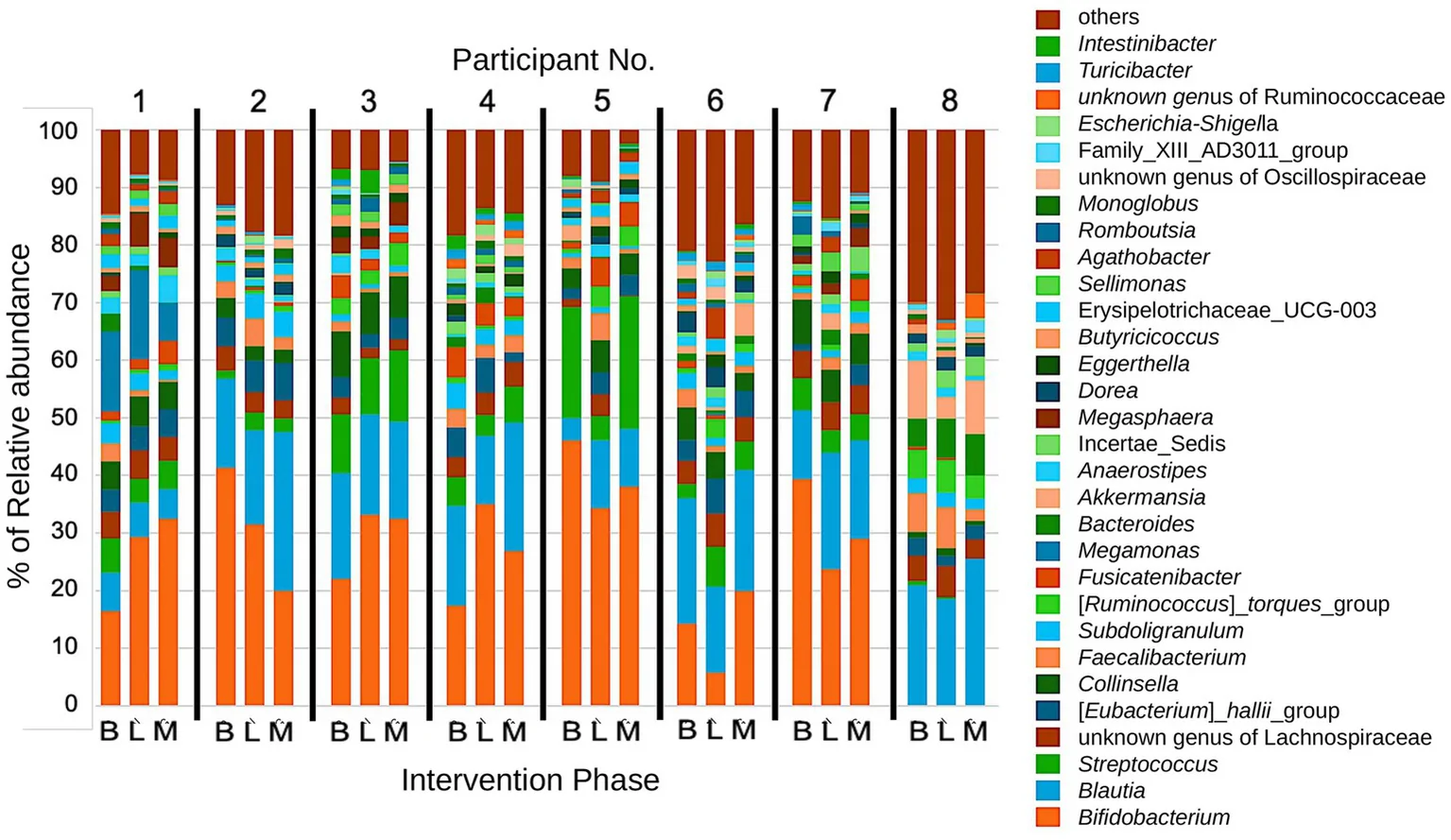
Genus-level gut microbiota composition across intervention phases. Stacked bar plots showing relative abundance of bacterial genera from eight participants during baseline (B), L-PDF (L), and M-PDF (M) phases. Each bar represents the mean taxonomic composition from three fecal samples per phase. Individual-specific compositional patterns are evident, with no consistent cohort-level directional changes.

Alpha diversity metrics showed participant-specific responses (Table 2; Supplementary Figures S4a–d). In these exploratory, per-participant comparisons—reported as nominal, uncorrected *p*-values—Chao1 richness showed the most pronounced changes (5/8 participants with nominally significant changes during M-PDF), whereas Shannon diversity exhibited minimal responsiveness (1/8 participants). Consistent with the group-level PERMANOVA (above), these individual-level fluctuations did not amount to a directional, cohort-level shift in community structure. Genus-level taxonomic analysis revealed individual-specific shifts without consistent cohort-level directionality (Figure 5). The most abundant genera across the cohort included *Bifidobacterium*, *Blautia*, *Faecalibacterium*, *Subdoligranulum*, *Bacteroides*, *Agathobacter*, *Anaerostipes*, and *Fusicatenibacter*. The direction and magnitude of change in these genera between phases differed from participant to participant, and none showed a consistent cohort-level increase or decrease during either intervention phase. Notably, even among the six participants receiving inulin as M-PDF, *Bifidobacterium* responses varied considerably, underscoring that functional capacity depends on factors beyond taxonomic composition alone.

**Table 2:** Summary of alpha- and beta-diversity metric responses by participant.

Diversity metric	1	2	3	4	5	6	7	8	Responders
Chao1 richness	↓**/↔	↔/↓*	↔/↑*	↔/↓**	↑**/↑**	↔/↔	↔/↑*	↔/↔	2/8 (L); 5/8 (M)
Shannon diversity	↓*/↓*	↔/↔	↔/↔	↔/↔	↔/↔	↔/↔	↔/↔	↔/↔	1/8 (L); 1/8 (M)
Observed features	↔/↓**	↔/↔	↔/↔	↔/↔	↔/↔	↔/↔	↔/↔	↔/↔	0/8 (L); 1/8 (M)
Weighted UniFrac	↔/↔	↑**/↑**	↔/↔	↑**/↔	↔/↔	↑*/↔	↔/↔	↔/↔	3/8 (L); 1/8 (M)

Direction of change is shown for each participant as BL → L-PDF/BL → M-PDF. Symbols: ↑** significant increase (*p* < 0.01); ↑* significant increase (*p* < 0.05); ↓** significant decrease (*p* < 0.01); ↓* significant decrease (*p* < 0.05); ↔ no substantial change. *p*-values were calculated using Kruskal–Wallis tests comparing baseline and intervention phases; these are exploratory per-participant comparisons, and asterisks denote nominal (uncorrected) significance that was not adjusted for multiple comparisons. The cohort-level assessment of community structure is based on PERMANOVA (see main text). BL, baseline; L-PDF/M-PDF, least/most prebiotic dietary fiber phase.

### 3.7 Prebiotic response spectra across intervention phases

**Figure 6:**
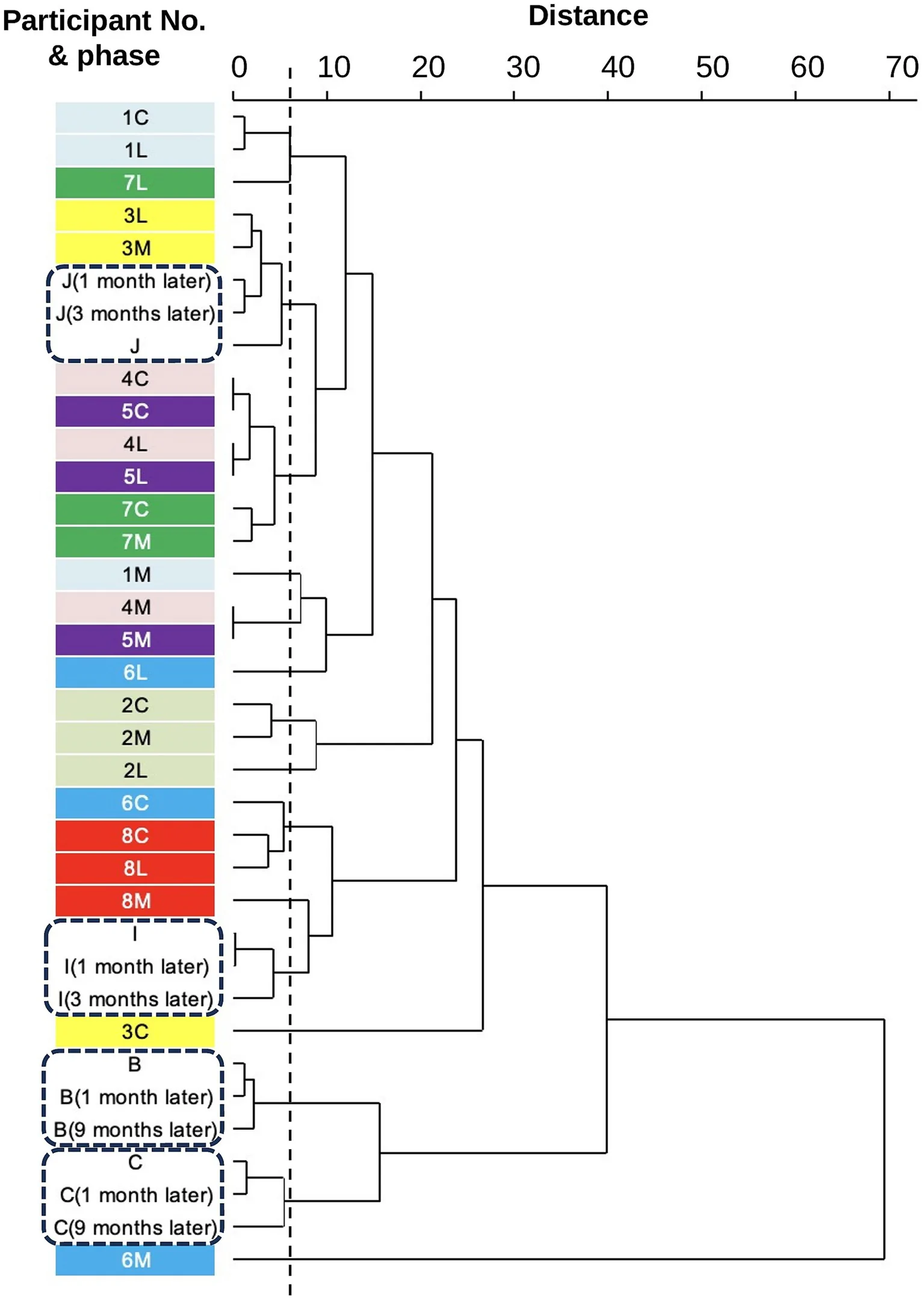
UPGMA dendrogram of prebiotic response spectra from validation and intervention cohorts. Hierarchical cluster analysis of prebiotic response profiles from both the validation study and intervention trial. For direct comparison, only the 13 commonly available dietary fiber products were analyzed. The distance scale (0–70) represents dissimilarity calculated using the Horn similarity index.

UPGMA clustering of prebiotic response spectra revealed that longitudinal validation samples formed tight individual-specific clusters (distances consistently below 7.0), while intervention participants showed more heterogeneous clustering patterns (Figure 6). The individual prebiotic response spectra measured during the intervention phases are shown in Supplementary Figure S5. Mean SI increased modestly in both intervention phases (L-PDF: +0.057 ± 0.051; M-PDF: +0.059 ± 0.042) with no significant inter-phase difference (paired *t*-test: mean difference = 0.002, 95% CI: −0.032–0.036, *p* = 0.91), indicating that neither intervention substantially shifted overall (average) fermentation capacity, even though individual response profiles could vary across phases.

## 4 Discussion

This study provides, to our knowledge, the first prospective, function-based proof-of-concept framework for personalized dietary fiber selection at the individual microbiome level. While the modest sample size and surrogate endpoint (fecal VFA) preclude definitive clinical conclusions, the directional response in seven of eight participants—each showing greater VFA production during their MyDFF-identified optimal fiber phase—constitutes preliminary proof-of-concept evidence that microbiome-guided fiber selection can offer functional advantages over non-personalized approaches. This directional consistency suggests that the advantage of MyDFF-guided selection reflects a response shared across most of the cohort rather than one driven by a few outliers, albeit in a small, exploratory sample. Importantly, one participant (No. 8) showed no advantage from the MyDFF-selected fiber, tempering any claim of universal efficacy. Nevertheless, the higher-ranked M-PDF was directionally concordant with the greater *in vivo* VFA response in seven of eight participants, although the magnitude of the *ex vivo* SI differential did not predict the magnitude of the *in vivo* response (see below).

Importantly, metabolic improvements occurred without major restructuring of microbial community composition. The dissociation between functional enhancement and compositional stability is a key conceptual finding of this study. Within the framework of functional redundancy in microbial ecology, multiple taxonomically distinct organisms may carry similar metabolic capabilities, such that shifts in community-level VFA output can occur through altered gene expression, substrate utilization priorities, or cross-feeding dynamics rather than through changes in dominant taxa. This interpretation is consistent with emerging concepts in gut microbiome ecology that emphasize functional capacity over taxonomic identity (32, 33). Importantly, this framing carries a practical implication: if meaningful metabolic benefits can be achieved without disrupting established microbial communities, precision prebiotic interventions may offer a more sustainable strategy than approaches that aim to reshape community composition. We note, however, that the 16S rRNA approach employed here cannot resolve the underlying mechanisms—metatranscriptomic and metabolomic profiling in future studies will be necessary to directly test whether altered gene expression or metabolic reallocation explains the observed functional gains.

The temporal stability of individual fermentation profiles over 3–9 months observed in the pre-intervention validation study is potentially relevant for clinical translation. In the four validation donors followed longitudinally (B, C, I, J), individual response profiles were maintained across timepoints, suggesting that a single screening may remain informative over this interval. We emphasize, however, that this observation rests on a small number of donors and a follow-up window of at most 9 months; it does not establish that a single screening remains valid indefinitely, and periodic re-assessment may be required, particularly after substantial dietary change, illness, or antibiotic exposure. Previous studies have established that gut microbiome composition is generally stable in healthy adults over similar timeframes (34, 35), and our data provide preliminary evidence extending this to functional fermentation phenotypes.

The strong correlation between SI values and SCFA concentrations measured in the same *ex vivo* fermentation assay (r^2^ = 0.79) supports the validity of our pH-based approach as a practical and scalable screening tool. *In vivo*, however, the *ex vivo* SI differential did not quantitatively predict the size of the VFA response differential (*r* = 0.082; *r* = 0.65, *p* = 0.11 when the single non-responder is excluded), indicating that although the assay reliably ranks fiber fermentability *in vitro*, the magnitude of the *in vivo* response is shaped substantially by host physiological factors—including gut transit time, absorption kinetics, and habitual dietary intake—and by inter-individual variability that a small cohort cannot resolve (36–38).

A notable finding was that four participants (50%) achieved VFA increases substantially exceeding theoretical yields from complete fermentation of the 6 g daily supplemented fiber (~30–48 μmol/g). Several non-mutually exclusive mechanisms may account for these supra-stoichiometric yields: MyDFF-selected fibers may have stimulated fermentation of endogenous dietary fiber already present in the gut (39), keystone fiber degraders may have established synergistic cross-feeding networks with downstream butyrate producers (40, 41), or provision of a preferred exogenous carbon source may have reduced reliance on colonic mucin as an alternative substrate. These hypotheses remain speculative and require direct validation through metagenomic and metabolomic approaches in future studies.

The divergent VFA responses among participants with similar SI values for inulin—and the variable *Bifidobacterium* responses among the six inulin-receiving participants despite its well-characterized bifidogenic properties (42)—underscore that neither SI alone nor taxonomic composition alone provides complete prediction. Future platform iterations should integrate complementary physiological parameters and potentially strain-level resolution to improve predictive accuracy.

Several limitations warrant acknowledgement. The sample size (*n* = 8) is modest; however, the crossover design with within-participant comparisons reduces the influence of stable between-participant variability on the primary comparison, and the directional consistency in seven of eight participants provides preliminary support for the observed effect that is not contingent on overall effect size alone (29). The *a priori* sample-size estimate was based on a within-participant baseline-versus-optimal-fiber comparison and on pre-study assumptions that proved smaller than the observed variability; the study was therefore not formally powered for the pre-specified per-participant Dunnett analysis, and all findings should be regarded as exploratory. In addition, intervention sequence was allocated by enrollment order rather than by formal randomization; although this produced an exactly balanced 4:4 distribution and preceded any knowledge of individual outcomes, we note this deviation from randomized allocation as a limitation. Although the paired crossover design reduces between-participant variability, potential sequence and period effects cannot be completely excluded. It should also be noted that habitual dietary intake and physical activity were not quantitatively recorded; although the within-participant crossover design controls for stable individual factors and monitoring captured fermentation-relevant supplements, medications, and antibiotics, unmeasured within-participant temporal variation cannot be fully excluded. Two participants (Nos. 7 and 8) received short antibiotic courses—during the washout and pre-observation periods, respectively—neither of which overlapped the fecal-sampling windows; because one was a responder and the other the non-responder, the available observations do not suggest a consistent directional association between antibiotic exposure and response. However, residual confounding and transient effects on individual fermentation profiles cannot be fully excluded.

Our cohort comprised healthy Japanese adults from a single geographic location, and generalizability beyond this population warrants explicit consideration. Japanese dietary patterns—characterized by high consumption of fermentable carbohydrates including rice starch and diverse seaweed-derived polysaccharides—may shape baseline microbiome composition and fermentation capacity in ways that differ from Western populations. Whether MyDFF-identified optimal fiber types for individuals with markedly different microbiome configurations (e.g., Western industrialized, rural African, or South Asian populations) would show comparable predictive validity is unknown. Similarly, disease states including inflammatory bowel disease, type 2 diabetes, or antibiotic-associated dysbiosis are likely to alter both fermentation profiles and their longitudinal stability, and the platform requires separate validation in clinical populations before therapeutic applications can be considered. Ethnic and geographic differences in microbiome composition are well-documented (43, 44), and multi-center trials in diverse cohorts are essential before generalizing the proof-of-concept findings reported here. The 6-week intervention duration may not capture long-term adaptation or durability of effects. We measured fecal VFA concentrations as a surrogate readout rather than clinical health endpoints; while this is standard practice in microbiome fermentation research, it means that clinical benefit remains to be established. Our 16S rRNA-based approach provides compositional profiles rather than metagenome-level functional information; the mechanisms underlying the taxon-function dissociation observed here—including potential roles of altered gene expression, cross-feeding, or substrate competition—cannot be resolved with the current data and await metatranscriptomic and metabolomic investigation.

The primary endpoint of this proof-of-concept trial was the change in fecal VFA concentration from baseline, which is a surrogate marker of gut microbial fermentative activity rather than a direct clinical outcome, and we acknowledge this as a limitation. Nevertheless, the biological plausibility that the observed VFA increases (mean difference 32.4 μmol/g; high-responders 110–121 μmol/g) contribute to host health is supported by evidence across multiple pathways (3–6): (i) epithelial barrier integrity and mucosal immunity through butyrate-mediated colonocyte energy supply and GPR41/43 signaling (4, 5); (ii) systemic metabolic effects via propionate-mediated hepatic gluconeogenesis and GLP-1/PYY enteroendocrine signaling (6); and (iii) anti-inflammatory effects through butyrate-mediated NF-κB inhibition (4, 5). Establishing causal links between the specific VFA increments observed here and clinical outcomes will require future trials powered to detect such endpoints, particularly in populations with metabolic syndrome, inflammatory bowel disease, or obesity where VFA optimization may confer the greatest therapeutic benefit.

The present study extends prior personalized prebiotic research in two important respects. First, whereas landmark studies have established retrospectively that individual identity is a stronger determinant of SCFA response than fiber type (15), they do not provide a prospective tool for fiber selection before intervention. MyDFF fills this gap by enabling *ex vivo* functional screening prior to supplementation, converting a retrospective observation into a prospective clinical decision. Second, our personalization approach is grounded in direct metabolic function rather than taxonomic composition. The finding that functional enhancement occurred without major compositional restructuring (PERMANOVA *p* > 0.6) is consistent with the concept that functional responses cannot be inferred solely from changes in taxonomic composition.

Future research priorities include larger multi-centre validation trials in diverse populations; evaluation in clinical conditions where VFA optimization may provide therapeutic benefit; integration of complementary omics approaches; and machine learning-based prediction models incorporating microbiome functional profiles, host physiology, and dietary background (45, 46).

In conclusion, matching dietary fiber to an individual’s own gut microbial fermentation capacity enhanced *in vivo* VFA production in most participants without restructuring the microbial community. Larger and more diverse cohorts, together with clinical endpoints, are now needed to determine whether this strategy translates into measurable health benefit.

## Data Availability

The datasets presented in this study can be found in online repositories. 16S rRNA gene sequencing data have been deposited in the DDBJ Sequence Read Archive under BioProject accession PRJDB40249 (Runs DRR904680-DRR904751). Short-chain fatty acid concentrations and saccharification index measurements are available via Zenodo: https://doi.org/10.5281/zenodo.18375790 (SCFA data); https://doi.org/10.5281/zenodo.18382490 (SI data, validation donors); and https://doi.org/10.5281/zenodo.18382675 (SI data, intervention participants). The code used for statistical analyses and data processing is available from the corresponding author upon reasonable request.
