# Supplementary material for: Function-based personalization of dietary fiber selection enhances gut microbial fermentation: a proof-of-concept crossover trial
Source: Front Nutr. 2026 Sep 3;13:1907988. doi: 10.3389/fnut.2026.1907988 (PMC13582658; doi:10.3389/fnut.2026.1907988)
Supplement: Supplementary file 1 [file Supplementary_file_1.docx]

**Table S1. Categories, sources, and chemical characteristics of dietary fiber substrates used in MyDFF analysis.**

| **Entry No.** | **Category** | **Derived or made from** | **Chemical features** |
| --- | --- | --- | --- |
| 1 | Resistant starch | Wheat starch | High-molecular carbohydrates composed of linear and branched α-1,4-d-glucan |
| 2 |  | Corn starch |  |
| 3 |  | Corn starch |  |
| 4 |  | Cassava starch |  |
| 5 | Resistant dextrin | Corn starch | A polymer of d-glucose units linked by α-1,2 and β-1,6 bonds |
| 6 |  | Corn starch |  |
| 7 |  | Corn starch |  |
| 8 | α-Cyclodextrin | Corn starch | A cyclic oligosaccharide of 6 glucose units linked by α-1,4 bonds |
| 9 |  | Corn starch |  |
| 10 |  | Corn starch |  |
| 11 | Isomaltodextrin | Corn starch | A polysaccharide made of d-glucose units linked by α-1,4 and α-1,6 glycosidic bonds of varying length |
| 12 |  | Unspecified starch |  |
| 13 | Partially hydrolyzed guar gum | Guar gum | Long, straight chains of α-D-mannopyranosyl units linked together by β-D-(1-4)-glycosidic linkage |
| 14 |  | Guar gum |  |
| 15 | Inulin | Chicory root | A linear fructosyl polymer linked by β-(2,1) bonds (n = 3–65), attached to a terminal glucosyl residue by an α-(1,2) bond |
| 16 |  | Enzymatically produced from sucrose |  |
| 17 |  | chicory root |  |

Comprehensive listing of 17 commercially available dietary fiber products used in the MyDFF screening panel, organized by six functional categories: resistant starch (Entry Nos. 1–4), resistant dextrin (5–7), α-cyclodextrin (8–10), isomaltodextrin (11–12), partially hydrolyzed guar gum (13–14), and inulin (15–17). For each product, the table includes entry number, fiber category, source, and key structural characteristics. During the intervention trial, four products (Entry Nos. 2, 4, 10, and 15) became temporarily unavailable due to supply issues or specification changes around January 2024. Complete product specifications including manufacturer and brand information are available upon reasonable request to maintain scientific objectivity and avoid commercial bias.

**Table S2. Fecal short-chain fatty acid concentrations across intervention phases in the personalized dietary fiber crossover trial.**

| **Participant No.** | **Testing phase** | **Acetate** | **Propionate** | **Butyrate** | **Lactate** | **Succinate** | **Total VFA** | **Total SCFA** |
| --- | --- | --- | --- | --- | --- | --- | --- | --- |
| 1 | BL | 98.6 | 26.4 | 7.9 | 0.0 | 2.2 | 132.9 | 135.1 |
|  | L-PDF | 106.8 | 28.6 | 10.6 | 3.9 | 3.2 | 146.0 | 153.1 |
|  | M-PDF | 149.5 | 29.9 | 16.7 | 0.0 | 4.5 | 196.1 | 200.6 |
| 2 | BL | 60.6 | 32.5 | 18.1 | 0.0 | 0.0 | 111.1 | 111.1 |
|  | L-PDF | 85.8 | 35.6 | 18.9 | 5.7 | 1.4 | 140.3 | 147.4 |
|  | M-PDF | 145.4 | 37.0 | 34.7 | 11.2 | 1.4 | 217.1 | 229.6 |
| 3 | BL | 67.5 | 31.2 | 17.8 | 0.0 | 2.0 | 116.5 | 118.5 |
|  | L-PDF | 105.4 | 32.3 | 28.6 | 0.0 | 0.0 | 166.4 | 166.4 |
|  | M-PDF | 158.5 | 35.9 | 32.1 | 0.0 | 0.0 | 226.6 | 226.6 |
| 4 | BL | 67.1 | 17.2 | 24.5 | 0.0 | 0.0 | 108.8 | 108.8 |
|  | L-PDF | 79.5 | 19.2 | 27.0 | 0.0 | 0.0 | 125.7 | 125.7 |
|  | M-PDF | 109.5 | 26.9 | 17.9 | 0.0 | 0.0 | 154.3 | 154.3 |
| 5 | BL | 97.2 | 22.5 | 18.4 | 14.9 | 0.0 | 138.2 | 153.1 |
|  | L-PDF | 114.7 | 32.7 | 23.2 | 0.0 | 0.0 | 170.6 | 170.6 |
|  | M-PDF | 152.9 | 24.8 | 26.0 | 5.5 | 0.0 | 203.7 | 209.2 |
| 6 | BL | 66.2 | 29.4 | 20.1 | 0.0 | 0.0 | 115.7 | 115.7 |
|  | L-PDF | 66.5 | 32.7 | 24.4 | 5.5 | 0.0 | 123.6 | 129.1 |
|  | M-PDF | 64.8 | 36.4 | 26.4 | 5.5 | 0.0 | 127.6 | 133.1 |
| 7 | BL | 41.9 | 20.1 | 18.7 | 0.0 | 0.0 | 80.7 | 80.7 |
|  | L-PDF | 49.1 | 22.3 | 20.6 | 0.0 | 0.0 | 92.0 | 92.0 |
|  | M-PDF | 55.8 | 23.6 | 19.4 | 0.0 | 0.0 | 98.8 | 98.8 |
| 8 | BL | 46.0 | 23.0 | 21.6 | 0.0 | 0.0 | 90.6 | 90.6 |
|  | L-PDF | 54.5 | 28.4 | 22.7 | 0.0 | 0.0 | 105.6 | 105.6 |
|  | M-PDF | 52.1 | 28.2 | 24.9 | 0.0 | 0.0 | 105.1 | 105.1 |

Individual and total short-chain fatty acid (SCFA) concentrations (µmol/g feces) quantified by HPLC in fecal samples from eight participants during baseline (BL), least prebiotic dietary fiber (L-PDF), and most prebiotic dietary fiber (M-PDF) intervention phases. Values represent mean concentrations from triplicate fecal samples collected during weeks 1–3 for baseline and weeks 4–6 for each intervention phase. Columns show individual volatile fatty acids (acetate, propionate, butyrate), non-volatile fatty acids (lactate, succinate), total VFA (sum of acetate, propionate, and butyrate), and total SCFA (sum of all five compounds). All units are µmol/g. VFAs comprised the predominant fraction (typically ≥90%) of total SCFAs. Statistical comparisons of VFA changes are presented in Figure 3A (mean difference = 32.4 µmol/g, 95% CI: 8.7–56.1 µmol/g, p = 0.014, Cohen’s d = 1.14). Corresponding individual saccharification indices are provided in main-text Table 1.

**Table S3. Pairwise PERMANOVA results based on weighted UniFrac distances among the eight participant groups.**

| **Group 1** | **Group 2** | **pseudo-F** | **p-value** | **q-value** |
| --- | --- | --- | --- | --- |
| 1 | 2 | 10.6598 | 0.001 | 0.00112 |
| 1 | 3 | 11.1793 | 0.001 | 0.00112 |
| 1 | 4 | 12.8802 | 0.001 | 0.00112 |
| 1 | 5 | 12.9817 | 0.001 | 0.00112 |
| 1 | 6 | 12.6569 | 0.001 | 0.00112 |
| 1 | 7 | 7.72548 | 0.001 | 0.00112 |
| 1 | 8 | 40.8936 | 0.001 | 0.00112 |
| 2 | 3 | 10.8141 | 0.001 | 0.00112 |
| 2 | 4 | 7.81784 | 0.001 | 0.00112 |
| 2 | 5 | 9.34219 | 0.001 | 0.00112 |
| 2 | 6 | 8.67752 | 0.001 | 0.00112 |
| 2 | 7 | 5.12935 | 0.001 | 0.00112 |
| 2 | 8 | 41.682 | 0.001 | 0.00112 |
| 3 | 4 | 12.5664 | 0.001 | 0.00112 |
| 3 | 5 | 4.23556 | 0.01 | 0.01 |
| 3 | 6 | 15.7437 | 0.001 | 0.00112 |
| 3 | 7 | 5.36829 | 0.003 | 0.003111 |
| 3 | 8 | 63.2372 | 0.001 | 0.00112 |
| 4 | 5 | 12.0442 | 0.001 | 0.00112 |
| 4 | 6 | 11.6544 | 0.001 | 0.00112 |
| 4 | 7 | 7.05887 | 0.001 | 0.00112 |
| 4 | 8 | 46.4182 | 0.001 | 0.00112 |
| 5 | 6 | 19.3417 | 0.001 | 0.00112 |
| 5 | 7 | 8.01154 | 0.002 | 0.002154 |
| 5 | 8 | 61.5091 | 0.001 | 0.00112 |
| 6 | 7 | 7.58908 | 0.001 | 0.00112 |
| 6 | 8 | 21.3165 | 0.001 | 0.00112 |
| 7 | 8 | 38.5271 | 0.001 | 0.00112 |

　Weighted UniFrac distances were computed from 72 fecal microbiome samples (nine samples per participant: three baseline, three L-PDF, and three M-PDF samples). Pairwise PERMANOVA tests were performed in QIIME 2 (999 permutations) to evaluate whether the between-group distances exceeded the within-group distances, indicating statistically significant separation between microbial communities. For each pair of participant groups (1–8), the table reports the PERMANOVA pseudo-F statistic, p-value, and FDR-corrected q-value. Significant differences (q < 0.05) indicate that the two groups have microbial community structures that are more dissimilar than would be expected from within-group variation. All 28 pairwise comparisons were significant (q < 0.05), demonstrating that participant identity is the dominant source of compositional variation. This finding is consistent with, and provides the pairwise basis for, the group-level result reported in the main text and shown in Fig. 4: samples cluster primarily by participant identity rather than intervention phase, with no significant shift in community structure following either L-PDF (p = 0.615) or M-PDF (p = 0.753) supplementation relative to baseline.

| **Participant No.** | **Questionnaires (n)** | **Antibiotics** | **Other medications / OTC** | **Non-study fiber supplements** | **Probiotic / fermented-dairy products** | **Other supplements / health foods** |
| --- | --- | --- | --- | --- | --- | --- |
| 1 | 9 | None | Rhinitis medication (occasional); eperisone and loxoprofen from mid-study | None | None | None |
| 2 | 9 | None | None | None | None | Daily multivitamin |
| 3 | 9 | None | OTC kampo (kakkonto) for a cold, a few days | None | None | None |
| 4 | 9 | None | None | None | None | None |
| 5 | 9 | None | None | None | None | None |
| 6 | 9 | None | Acetaminophen 500 mg, single day | None | None | Vitamin B complex, several days |
| 7 | 9 | Cefcapene pivoxil, 1-week course (unrelated injury) | None | None | None | None |
| 8 | 9 | Cefcapene pivoxil, 1-week course (pre-observation; via correspondence) | None | None | Probiotic fermented-dairy products (intermittent) | Zinc, iron, multivitamin, soy protein (habitual); St. John’s wort (daily) |

**Table S4. Self-reported intercurrent medication, supplement, and health-food use during the intervention.**

Scheduled physician interviews (nine questionnaires per participant) across the entire study period, including baseline and washout. Entries are anonymized self-reports; no assessment of each item’s effect on VFA production was performed. Habitual total dietary intake and physical activity were not systematically recorded. Participants 7 and 8 each received a 1-week oral cephalosporin (cefcapene pivoxil) course—participant 7 during the washout period and participant 8 during the pre-observation period (the latter was identified through study correspondence rather than the scheduled physician interviews; the baseline MyDFF screening was performed after the course had ended). Neither course overlapped the fecal-sampling windows used for VFA quantification. Participant 7 was among the seven responders and participant 8 was the sole non-responder; participant 8 also intermittently consumed probiotic fermented-dairy products.

**Figure S1. Correlation between Saccharification Index and total short-chain fatty acid production.**

Scatter plot showing the relationship between Saccharification Index (SI) values (x-axis) and HPLC-quantified total SCFA concentrations (y-axis, mM) in spent culture media from 30 randomly selected MyDFF wells spanning the full range of observed pH changes. Each point represents one well. The solid line shows linear regression fit with 95% confidence interval (shaded area). Strong positive correlation validates SI as a reliable proxy for microbial fermentation capacity (Pearson's r = 0.89, r² = 0.79, p < 0.01). This validation supports the use of pH-based SI measurements as a high-throughput screening approach for assessing ex vivo fermentation capacity.

**Figure S2. Individual prebiotic response spectra demonstrate inter-individual variability.**

Saccharification Index (SI) profiles for fecal microbiota from ten validation study donors (A-J) showing responses to 17 dietary fibers across six categories: resistant starch (Entry Nos. 1–4), resistant dextrin (5–7), α-cyclodextrin (8–10), isomaltodextrin (11–12), partially hydrolyzed guar gum (13–14), and inulin (15–17). X-axis shows dietary fiber entry numbers (see Table S1 for complete fiber identities and categories); y-axis shows SI values quantifying fermentation intensity (ΔpH between fiber-supplemented and control wells). Each bar represents mean ± SEM from triplicate measurements. Distinct patterns are evident: broad responders (Donors A, C, F, G, H, I, J) show elevated SI across multiple fiber categories, while narrow responders (Donors B, D, E) demonstrate selective substrate utilization with lower overall fermentation capacity. These individual-specific patterns correspond to the clustering analysis in Fig. 2.

**Figure S3. Longitudinal stability of prebiotic response spectra in validation donors.**

Temporal changes in Saccharification Index (SI) profiles for four validation study donors (B, C, I, J) showing prebiotic responses to 17 dietary fibers over time. Blue bars represent baseline samples, orange bars represent 1-month follow-up samples, and green bars represent long-term follow-up samples (3 months for donors I and J; 9 months for donors B and C). Each bar represents mean ± SEM from triplicate measurements. The x-axis shows dietary fiber entry numbers (see Table S1 for fiber identities and categories), and the y-axis represents SI values quantifying dietary fiber fermentation intensity by fecal microbiota. Individual response patterns remain relatively similar across timepoints, with broadly maintained rank order of fiber responses. These data support the temporal stability of individual fermentation profiles over the observed 3–9-month follow-up period in the small validation subset analyzed in Fig. 2.

**Figure S4a. Changes in Chao1 richness index of fecal microbiota from eight participants throughout the intervention study.**

Bar plots showing Chao1 richness estimates for each participant across study phases. Chao1 was computed separately from the rarefied dataset (rarefaction depth: 5,000 sequences per sample) and estimates total species richness including unseen species. Data represent mean values ± SEM from triplicate fecal samples collected during baseline (B, blue bars), L-PDF intervention (L, orange bars), and M-PDF intervention (M, green bars) phases. Statistical comparisons were performed in GraphPad Prism (version 9.5.1) using one-way ANOVA (or Welch's ANOVA when variances were unequal), or Kruskal-Wallis tests when parametric assumptions were not met, with Dunn's or Dunnett's post-hoc tests applied as appropriate, accounting for the repeated-measures design. P-values are displayed above brackets for significant comparisons (P < 0.05). Notable findings: 5 of 8 participants (63%) showed significant changes during M-PDF, with individual-specific directionality (some increases, some decreases), highlighting heterogeneous responses to personalized fiber interventions. P-values shown above are nominal (uncorrected) values from exploratory per-participant comparisons; given the large number of individual-level tests in a small cohort, they were not adjusted for multiple comparisons and are interpreted descriptively. The cohort-level assessment of community structure is based on the group-level PERMANOVA (see Fig. 4 and main-text Statistical analyses).

**Figure S4b. Changes in Shannon index of fecal microbiota from eight participants throughout the intervention study.**

Bar plots showing Shannon diversity index for each participant across study phases. Shannon diversity was calculated from rarefied data (rarefaction depth: 5,000 sequences per sample) and quantifies both species richness and evenness, with higher values indicating greater diversity. Data represent mean values ± SEM from triplicate fecal samples collected during baseline (B, blue bars), L-PDF intervention (L, orange bars), and M-PDF intervention (M, green bars) phases. Statistical comparisons were performed in GraphPad Prism (version 9.5.1) using one-way ANOVA (or Welch's ANOVA when variances were unequal), or Kruskal-Wallis tests when parametric assumptions were not met, with Dunn's or Dunnett's post-hoc tests applied as appropriate, accounting for the repeated-measures design. P-values are displayed above brackets for significant comparisons (P < 0.05). Only participant 1 showed significant changes (decreases during both L-PDF and M-PDF, both p < 0.05), while remaining participants maintained stable Shannon diversity across interventions. P-values above are nominal (uncorrected) values from exploratory per-participant comparisons and were not adjusted for multiple comparisons; the cohort-level assessment of community structure is based on the group-level PERMANOVA (see Fig. 4).

**Figure S4c. Changes in Observed features index of fecal microbiota from eight participants throughout the intervention study.**

Bar plots showing observed ASV (amplicon sequence variant) counts for each participant across study phases. Observed features were calculated from rarefied data (rarefaction depth: 5,000 sequences per sample) and represent the actual number of distinct ASVs detected in each sample. Data represent mean values ± SEM from triplicate fecal samples collected during baseline (B, blue bars), L-PDF intervention (L, orange bars), and M-PDF intervention (M, green bars) phases. Statistical comparisons were performed in GraphPad Prism (version 9.5.1) using one-way ANOVA (or Welch's ANOVA when variances were unequal), or Kruskal-Wallis tests when parametric assumptions were not met, with Dunn's or Dunnett's post-hoc tests applied as appropriate, accounting for the repeated-measures design. P-values are displayed above brackets for significant comparisons (P < 0.05). Minimal responsiveness overall, with only participant 1 showing significant decrease during M-PDF (p < 0.05), indicating stable taxonomic richness despite functional changes in VFA production. P-values above are nominal (uncorrected) values from exploratory per-participant comparisons and were not adjusted for multiple comparisons; the cohort-level assessment of community structure is based on the group-level PERMANOVA (see Fig. 4).

**Figure S4d. Changes in Weighted Unifrac Distance of fecal microbiota from eight participants throughout the intervention study.**

Bar plots showing weighted UniFrac distances from baseline for each participant. Weighted UniFrac distances were calculated from rarefied data (rarefaction depth: 5,000 sequences per sample) and quantify compositional dissimilarity accounting for phylogenetic relationships and relative abundances, with higher values indicating greater community structure changes from baseline. Data represent mean values ± SEM from triplicate fecal samples collected during baseline (B, blue bars), L-PDF intervention (L, orange bars), and M-PDF intervention (M, green bars) phases. Statistical comparisons were performed in GraphPad Prism (version 9.5.1) using one-way ANOVA (or Welch's ANOVA when variances were unequal), or Kruskal-Wallis tests when parametric assumptions were not met, with Dunn's or Dunnett's post-hoc tests applied as appropriate, accounting for the repeated-measures design. P-values are displayed above brackets for significant comparisons (P < 0.05). Moderate compositional shifts evident in 3 of 8 participants during L-PDF and 1 of 8 during M-PDF, though group-level PERMANOVA showed no significant changes (see Fig. 4).

**Figure S5. Individual prebiotic response spectra across intervention phases.**

Saccharification Index (SI) profiles for 8 intervention participants showing responses to dietary fibers across study phases. Participants Nos. 1–3 were screened with 17 products, while participants Nos. 4–8 were screened with 13 products due to temporary unavailability of four products (Entry Nos. 2, 4, 10, and 15) at their respective enrollment times, resulting in gaps in the x-axis for these participants. Blue, orange, and green bars represent mean SI values (±SEM) from three fecal samples collected during baseline (BL), least prebiotic dietary fiber (L-PDF), and most prebiotic dietary fiber (M-PDF) phases, respectively. Dietary fiber accession numbers highlighted with orange boxes indicate the L-PDF prescribed to each participant; green boxes indicate the M-PDF prescribed to each participant. The x-axis shows dietary fiber accession numbers (see Table S1 for fiber identities). These individual spectra correspond to the clustering analysis shown in Fig. 6.
