# Supplementary material for: Function-based personalization of dietary fiber selection enhances gut microbial fermentation: a proof-of-concept crossover trial
Source: Front Nutr. 2026 Sep 3;13:1907988. doi: 10.3389/fnut.2026.1907988 (PMC13582658; doi:10.3389/fnut.2026.1907988)
Supplement: Supplementary file 2 [file Supplementary_file_2.docx]

**Supplementary Material**

**Function-based personalization of dietary fiber selection enhances gut microbial fermentation: a proof-of-concept crossover trial**

Ro Osawa, Jun Inoue, Itsuko Fukuda, Yasuhito Shirai, Yuzo Kodama

*** Correspondence:** Ro Osawa, koolkoala@topaz.kobe-u.ac.jp

**Supplementary Methods**

**S1. MyDFF dietary fiber panel**

The dietary fiber panel comprised 17 commercially available supplements representing six categories: resistant starch (n = 4), resistant dextrin (n = 3), α-cyclodextrin (n = 3), isomaltodextrin (n = 2), partially hydrolyzed guar gum (n = 2), and inulin (n = 3). Product details are summarized in Supplementary Table S1. Full manufacturer and brand information is available upon reasonable request to maintain scientific objectivity. During the intervention trial, four products (Entry Nos. 2, 4, 10, and 15) became temporarily unavailable; participants enrolled after January 2024 (Nos. 4–8) were therefore screened with a 13-product panel.

**S2. Ex vivo fermentation assay**

Individual fibers were added at 1% (w/v) final concentration to 1 mL of modified Gifu Anaerobic Medium^S1^ in 96-well format. Each well was inoculated with 50 µL of fecal suspension (~10^8^ cells/mL) prepared from fresh fecal samples and incubated anaerobically (AnaeroPack system; Mitsubishi Gas Chemical Company, Tokyo, Japan) at 37 °C for 24 h. Post-incubation pH was measured using a microelectrode (ToupH 9669-10D; HORIBA Advanced Techno, Kyoto, Japan). All fermentations were performed in triplicate. The saccharification index (SI) was calculated as the mean ΔpH between fiber-supplemented and fiber-free control wells: SI = pH_control_ − pH_fiber_. Higher SI values indicate greater saccharolytic (fermentative) activity.

**S3. SCFA/VFA quantification by HPLC**

Thawed fecal samples (approximately 0.1 g) were homogenized in 0.9 mL of 0.15 mol/L phosphoric acid solution and centrifuged at 20,000 × *g* for 15 min at 4 °C. The supernatant was filtered through a 0.45-µm membrane and analyzed by high-performance liquid chromatography (HPLC) using an Aminex HPX-87H ion-exclusion column (Bio-Rad Laboratories, Hercules, CA, USA) with refractive index detection (RID-10A; Shimadzu Corporation, Kyoto, Japan). The mobile phase was 5 mmol/L H_2_SO_4_ at a flow rate of 0.6 mL/min at 65 °C. Volatile fatty acids (acetate, propionate, butyrate) and non-volatile fatty acids (lactate, succinate) were quantified against external standards. Total VFA was defined as the sum of acetate, propionate, and butyrate; total SCFA as the sum of all five compounds.

**S4. 16S rRNA gene sequencing**

Bacterial genomic DNA was extracted from thawed fecal samples (~0.2 g) using a bead-beating protocol with the QIAamp PowerFecal Pro DNA Kit (Qiagen, Hilden, Germany). The V3–V4 hypervariable regions of the 16S rRNA gene were amplified using universal primers S-D-Bact-0341-b-S-17 (5′-CCTACGGGNGGCWGCAG-3′) and S-D-Bact-0785-a-A-21 (5′-GACTACHVGGGTATCTAATCC-3′). Paired-end sequencing (2 × 300 bp) was performed on an Illumina MiSeq platform using MiSeq Reagent Kit v3 (Illumina, San Diego, CA, USA) with PhiX Control v3 as an internal standard. Demultiplexed paired-end reads, from which primer sequences had been removed during MiSeq preprocessing, were imported into QIIME 2 (version 2025.7) and processed using the DADA2 denoise-paired method. Diversity analyses used a rarefaction depth of 5,000 sequences per sample. Alpha diversity metrics (Chao1, Shannon, observed ASVs) were computed from the rarefied dataset. Beta diversity was assessed using weighted UniFrac distances with principal coordinates analysis (PCoA) ordination. Among the 72 samples included in the microbiome analyses, the number of input read pairs ranged from 81,386 to 1,401,550 per sample (mean, 151,684; median, 133,526). Denoising parameters were as follows: trim-left 0/0; trunc-len 285/230; trunc-q 2; maxEE 2.0/3.0; n-reads-learn 10,000.

Following quality filtering, denoising, paired-end merging, and consensus chimera removal, 5,800–75,361 non-chimeric sequences per sample remained (mean, 12,692; median, 11,989). The final feature table contained 6,419 DADA2-generated amplicon sequence variants (ASVs). No samples were excluded because of sequencing failure, DADA2 quality-control failure, or insufficient sequencing depth. For phylogenetic analyses, representative ASVs were aligned with MAFFT, hypervariable positions were masked, and a midpoint-rooted FastTree was constructed within QIIME 2. Taxonomy was assigned using q2-feature-classifier with a pre-trained naïve Bayes classifier trained on the SILVA version 138.1 SSURef NR99 reference dataset (confidence threshold 0.7); no fixed sequence-similarity threshold was applied, consistent with the exact-sequence resolution of DADA2-generated ASVs. The rarefaction depth of 5,000 sequences per sample was selected because the minimum post-DADA2 sequence count across all 72 samples was 5,800 (allowing all samples to be retained), and because alpha-rarefaction curves showed diminishing gains in the principal alpha-diversity metrics at approximately this depth.

**S5. Statistical analyses**

The primary endpoint was the per-participant change in fecal short-chain fatty acid concentrations between baseline and each intervention phase. For each participant, the three fecal samples collected per phase were compared across phases (baseline, L-PDF, M-PDF) by one-way ANOVA with Dunnett's multiple-comparison test (L-PDF vs. baseline; M-PDF vs. baseline) in Statcel2, for both total VFA and total SCFA. As a post hoc supportive analysis at the cohort level, baseline-referenced contrasts (L-PDF vs. baseline; M-PDF vs. baseline) for total VFA were evaluated with a linear mixed-effects model fitted to all 72 measurements, with study phase as a fixed effect and participant as a random intercept (restricted maximum likelihood; Kenward–Roger degrees of freedom; emmeans package), and the two baseline-referenced contrasts were adjusted by a Dunnett-type procedure with multivariate-t adjustment. The protocol-specified within-participant comparison (M-PDF vs. L-PDF ΔVFA) used a two-tailed paired t-test. The probability of observing seven of eight participants responding in the same direction by chance was assessed in a post hoc exploratory analysis using a two-sided binomial test (null probability 0.5 per participant, 8 independent observations). Group-level differences in gut microbiota composition were evaluated by PERMANOVA with weighted UniFrac distances (999 permutations). Alpha diversity comparisons across phases used one-way ANOVA (or Welch’s ANOVA when variances were unequal) or Kruskal–Wallis tests when parametric assumptions were not met, with post-hoc testing as appropriate. Hierarchical clustering of prebiotic response spectra used the UPGMA algorithm with the Horn similarity indexS2,S3 implemented in R (v4.3.0). Pearson correlation was used to assess SI–SCFA and ΔSI–ΔVFA relationships. All tests were two-sided; p < 0.05 was considered statistically significant. Effect sizes are reported as Cohen’s d. Statistical analyses were performed in Statcel2, R (v4.3.0), GraphPad Prism (v9.5.1), and Python (v3.11).

Multiple comparison considerations: (1) the primary per-participant baseline-referenced comparisons (L-PDF vs. BL; M-PDF vs. BL) used Dunnett's test (Statcel2), which controls the family-wise error rate across the two simultaneous comparisons against the common baseline within each participant; (2) the protocol-specified within-participant paired t-test (M-PDF vs. L-PDF ΔVFA) constitutes a single test and requires no further multiplicity adjustment; (3) the exact binomial test of directional concordance was a post hoc exploratory analysis and was interpreted descriptively; (4) the per-participant alpha-diversity comparisons (Chao1, Shannon, observed ASVs; Figures S4a–d) are exploratory and are reported as nominal (uncorrected) p-values; given the large number of individual-level tests in a small cohort, formal family-wise correction was not applied and these comparisons are interpreted descriptively. The confirmatory assessment of community structure is based on the group-level PERMANOVA of weighted UniFrac distances.

**Supplementary References**

S1. Gotoh A, et al. Use of Gifu Anaerobic Medium for culturing 32 dominant species of human gut microbes. *Biosci Biotechnol Biochem* (2017) 81:2009–2017. doi: 10.1080/09168451.2017.1359486

S2. Chao A, et al. Abundance-based similarity indices and their estimation when there are unseen species in samples. *Biometrics* (2006) 62:361–371. doi: 10.1111/j.1541-0420.2005.00489.x

S3. Gloor GB, et al. Microbiome datasets are compositional: and this is not optional. *Front Microbiol* (2017) 8:2224. doi: 10.3389/fmicb.2017.02224
